# Endocardial Left Atrial Appendage Occlusion in Atrial Fibrillation: Device Design, Clinical Evidence, and Postprocedural Antithrombotic Management

**DOI:** 10.3390/medicina62091644

**Published:** 2026-08-27

**Authors:** Grigory Matvienko, Mantė Agnė Rimkienė, Vilhelmas Bajoras, Agnė Drąsutienė, Rūta Masiulienė, Dovilė Gabartaitė, Monika Šalaševičienė, Mindaugas Matačiūnas, Diana Sudavičienė, Germanas Marinskis, Audrius Aidietis, Gediminas Račkauskas

**Affiliations:** 1 Clinic of Cardiac and Vascular Diseases, Institute of Clinical Medicine, Faculty of Medicine, Vilnius University, 03101 Vilnius, Lithuania; mante.rimkiene@santa.lt (M.A.R.); vilhelmas.bajoras@santa.lt (V.B.); agne.drasutiene@santa.lt (A.D.); monika.salaseviciene@santa.lt (M.Š.); diana.sudaviciene@santa.lt (D.S.); germanas.marinskis@santa.lt (G.M.); audrius.aidietis@santa.lt (A.A.); gediminas.rackauskas@santa.lt (G.R.); 2 Center of Cardiology and Angiology, Vilnius University Hospital Santaros Klinikos, 08406 Vilnius, Lithuania; ruta.masiuliene@santa.lt (R.M.); dovile.gabartaite@santa.lt (D.G.); 3 Department of Radiology, Nuclear Medicine and Medical Physics, Institute of Biomedical Sciences, Faculty of Medicine, Vilnius University, 01513 Vilnius, Lithuania; mindaugas.mataciunas@santa.lt

**Keywords:** left atrial appendage occlusion, left atrial appendage closure, atrial fibrillation, stroke prevention, endocardial devices, device design, postprocedural antithrombotic therapy, device-related thrombus, peri-device leak, direct oral anticoagulants

## Abstract

*Background and Objectives:* Atrial fibrillation is a major cause of ischemic stroke, and in patients with non-valvular atrial fibrillation the left atrial appendage is the predominant site of thrombus formation. Although oral anticoagulation remains the standard strategy for stroke prevention, long-term therapy may be limited by bleeding risk, prior major bleeding, contraindications, intolerance, or difficulty maintaining stable anticoagulation. Endocardial left atrial appendage occlusion (LAAO) has therefore emerged as an alternative strategy in selected patients. Its role continues to evolve with expanding device technologies, contemporary direct oral anticoagulant therapy, and ongoing randomized evidence. *Materials and Methods:* This narrative literature review summarizes current evidence on endocardial LAAO, focusing on device design, anatomical considerations, clinical outcomes, postprocedural antithrombotic therapy, and ongoing research directions. *Results:* Available devices differ substantially in sealing mechanism, fixation strategy, conformability, surface properties, and suitability for complex appendage anatomy. The Watchman and Amulet platforms remain the most extensively studied systems, whereas several CE-marked and investigational devices aim to improve anatomical adaptability, reduce peri-device leak and device-related thrombus, and simplify implantation. Randomized evidence supports LAAO as a non-inferior alternative to oral anticoagulation in selected populations, with potential reduction in late bleeding but persistent concerns regarding procedural risk, frailty, comorbidity burden, and patient selection. The optimal postprocedural antithrombotic regimen remains uncertain, with increasing interest in individualized strategies and short-term direct oral anticoagulant therapy when feasible. Current research is shifting toward next-generation devices, combined ablation–closure strategies, simplified imaging guidance, and evaluation of LAAO as both an alternative and an adjunct to oral anticoagulation. *Conclusions:* LAAO should currently be considered a selective rather than a universal strategy, requiring individualized assessment of thromboembolic risk, bleeding risk, anatomy, procedural safety, and comorbidity burden.

## 1. Introduction

Atrial fibrillation (AF) is the most common sustained cardiac arrhythmia and a major contributor to ischemic stroke, heart failure, mortality, and healthcare utilization. Its prevalence increases substantially with age, making AF-related thromboembolism an increasingly important clinical challenge in ageing populations [1]. Although the mechanisms underlying thrombus formation in AF are multifactorial, left atrial mechanical dysfunction, blood stasis, endothelial dysfunction, and systemic prothrombotic changes collectively promote intracardiac thrombogenesis. Within this pathophysiological framework, the left atrial appendage (LAA) has particular clinical relevance. Owing to its variable anatomy, narrow orifice, trabeculated structure, and low-flow environment, the LAA represents the predominant site of thrombus formation in non-valvular AF, with approximately 90% of left atrial thrombi reported to originate from this structure [2,3,4].

Oral anticoagulation remains the cornerstone of stroke prevention in AF [1,5]. The introduction of direct oral anticoagulants (DOACs) has reshaped clinical practice by reducing several practical and safety limitations of vitamin K antagonist therapy, including routine coagulation monitoring, dietary restrictions, and variability in anticoagulant effect [1,6]. However, DOACs have not eliminated the clinical problem of patients in whom sustained systemic anticoagulation is contraindicated, poorly tolerated, or associated with an unfavorable net clinical benefit [1,5,7]. This population is heterogeneous and may include patients with prior major bleeding, nonreversible bleeding predisposition, severe frailty, advanced renal or hepatic dysfunction, or complex comorbidity burden. In such patients, stroke prevention requires an individualized strategy that balances thromboembolic risk against bleeding risk, procedural risk, and life expectancy. These limitations have provided the principal rationale for left atrial appendage occlusion (LAAO) as a targeted, non-pharmacological approach to stroke prevention in carefully selected patients [1,5,7].

The field of LAAO has evolved rapidly from early endocardial occluders to a diverse landscape of contemporary and investigational devices [6,8,9]. Current platforms differ substantially in their sealing mechanism, anchoring strategy, implantation depth, surface characteristics, conformability, and interaction with complex LAA anatomy. These design features are not merely technical attributes; they may influence implantation success, residual peri-device leak, device-related thrombus, endothelialization, and the need for postprocedural antithrombotic therapy [8,9,10,11,12,13]. At the same time, the clinical evidence base has expanded from early warfarin-comparator trials to contemporary randomized and registry-based data evaluating LAAO in the era of DOAC therapy [14,15,16,17,18,19]. Nevertheless, important uncertainties remain regarding appropriate patient selection, comparative effectiveness against contemporary anticoagulation, and optimal postprocedural antithrombotic management.

This narrative review summarizes contemporary and emerging endocardial LAAO devices, focusing on their design principles, clinical evidence, and postprocedural antithrombotic management. Particular attention is given to the potential interaction between device-related characteristics, anatomical suitability, patient risk profile, and antithrombotic strategy. Surgical and epicardial approaches are beyond the scope of this review.

## 2. Materials and Methods

This article was designed as a narrative review using an iterative, device-centered literature search. Initially, published narrative and systematic reviews of endocardial left atrial appendage occlusion (LAAO) were examined to identify contemporary and emerging devices. Identified devices were recorded in a working database and subsequently searched individually in PubMed/MEDLINE, ClinicalTrials.gov, Google Scholar, and Google using combinations of the device name with terms including “LAAO”, “left atrial appendage occlusion”, and “left atrial appendage device”. When additional devices were identified during screening, they were added to the database and searched using the same approach. Searches were updated through 30 April 2026.

Because published evidence for several emerging devices was limited, all relevant English-language full-text publications mentioning these devices were considered for further evaluation. Clinical trials, observational studies, registries, reviews, guidelines, regulatory sources, and relevant preclinical studies were included according to the topic being assessed. Conference abstracts and trial registrations were used primarily to identify emerging technologies and ongoing studies. Surgical and epicardial LAA exclusion techniques were outside the primary scope of the review.

In addition to device-specific searches, targeted searches were performed for topics required for the clinical synthesis, including randomized evidence, comparative effectiveness, device-related thrombus, peri-device leak, imaging, and postprocedural antithrombotic management. These searches were intended to identify the most relevant available evidence rather than to constitute a systematic comparative search of all LAAO studies. After removal of duplicates, 303 records were retained for screening. Study selection was performed collaboratively by two authors; screening was not conducted as two independent parallel assessments. Relevant sources were screened by title and abstract; potentially relevant publications were assessed in full text, and study and device information was recorded in a structured working table. Evidence was synthesized qualitatively, with randomized and prospective clinical evidence distinguished from observational, developmental, regulatory, imaging, and preclinical or mechanistic evidence. No formal risk-of-bias assessment or meta-analysis was performed.

## 3. Results

### 3.1. Rationale for LAAO and Current Guideline Positioning

#### 3.1.1. Limitations of Long-Term Oral Anticoagulation

Despite its central role in stroke prevention, long-term oral anticoagulation may be difficult to maintain or clinically unfavorable in selected patients. Relevant limitations include prior major bleeding, persistent or non-reversible bleeding predisposition, advanced frailty or competing comorbidity burden, severe renal or hepatic dysfunction, clinically important drug interactions, poor adherence, and inability to maintain stable vitamin K antagonist (VKA) therapy [1,5,7].

Bleeding risk is commonly assessed using clinical scores such as HAS-BLED, HEMORR_2_HAGES, or ATRIA. These tools are useful for identifying patients who require closer follow-up and correction of modifiable bleeding risk factors, but they should not be interpreted as stand-alone instruments for withholding anticoagulation. A high bleeding risk score does not, by itself, constitute an absolute contraindication to oral anticoagulation and should be considered together with thromboembolic risk, comorbidity burden, patient preferences, and the feasibility of risk-factor modification [1,5].

In clinical practice, the distinction between modifiable, partially modifiable, and non-modifiable bleeding risk factors is essential. Whenever possible, reversible contributors should be addressed, including uncontrolled hypertension, concomitant antiplatelet or non-steroidal anti-inflammatory drug use, excessive alcohol intake, labile INR, or avoidable drug interactions [1,5]. In contrast, patients with non-reversible bleeding predisposition, recurrent major bleeding, or comorbidity profiles associated with an unfavorable net clinical benefit from long-term anticoagulation may require alternative stroke prevention strategies [1,5,7]. Common clinical scenarios that may limit long-term oral anticoagulation are summarized in Table 1.

These limitations do not diminish the central role of anticoagulation in AF but highlight the need for individualized decision-making. In carefully selected patients in whom long-term anticoagulation is contraindicated, poorly tolerated, or clinically unfavorable, LAAO may be considered as a non-pharmacological alternative for stroke prevention [1,5,7].

#### 3.1.2. Guideline Positioning

Contemporary guidelines position LAAO as a selective, non-pharmacological option for stroke prevention in patients with AF in whom long-term oral anticoagulation is contraindicated, poorly tolerated, or associated with an unfavorable risk–benefit profile [1,5,7]. However, recommendations remain cautious and emphasize individualized decision-making, reflecting heterogeneity in the available evidence and limited randomized data in patients who are truly unsuitable for long-term anticoagulation.

The 2023 ACC/AHA/ACCP/HRS guideline provides the strongest recommendation among current major guidelines. Percutaneous LAAO is considered reasonable in patients with AF, moderate-to-high thromboembolic risk, and a nonreversible contraindication to long-term oral anticoagulation. In patients with a high risk of major bleeding on oral anticoagulation, LAAO may be considered as an alternative after careful consideration of procedural risk and patient preference [5].

The 2024 ESC guideline gives a more conservative recommendation, stating that percutaneous LAAO may be considered in patients with AF and contraindications to long-term anticoagulant therapy. The guideline also emphasizes that bleeding risk should be systematically assessed and modifiable bleeding risk factors corrected whenever possible, rather than using bleeding risk alone as a reason to withhold anticoagulation [1].

The 2025 SCAI/HRS clinical practice guideline similarly supports transcatheter LAAO in selected patients with non-valvular AF who are not suitable candidates for long-term oral anticoagulation. This conditional recommendation highlights the importance of shared decision-making, appropriate procedural suitability, and individualized assessment of thromboembolic and bleeding risks [7].

Taken together, current guideline recommendations support LAAO as an alternative strategy for carefully selected patients rather than as a routine replacement for oral anticoagulation. The recommendations are summarized in Table 2 [1,5,7].

### 3.2. Device Design Principles and Classification

#### 3.2.1. Device Design Principles

Endocardial LAAO devices are intended to exclude the left atrial appendage from the left atrial cavity by creating a mechanical barrier between the appendage and systemic circulation. In most systems, the device is delivered through a transseptal approach and positioned either within the appendage body, across the ostium, or at both levels, depending on the device architecture [8,9]. The clinical objective is not simply to “fill” the appendage, but to achieve durable appendage exclusion while preserving device stability, minimizing residual flow, and limiting thrombogenic surface exposure [8,9,12].

**Figure 1:**
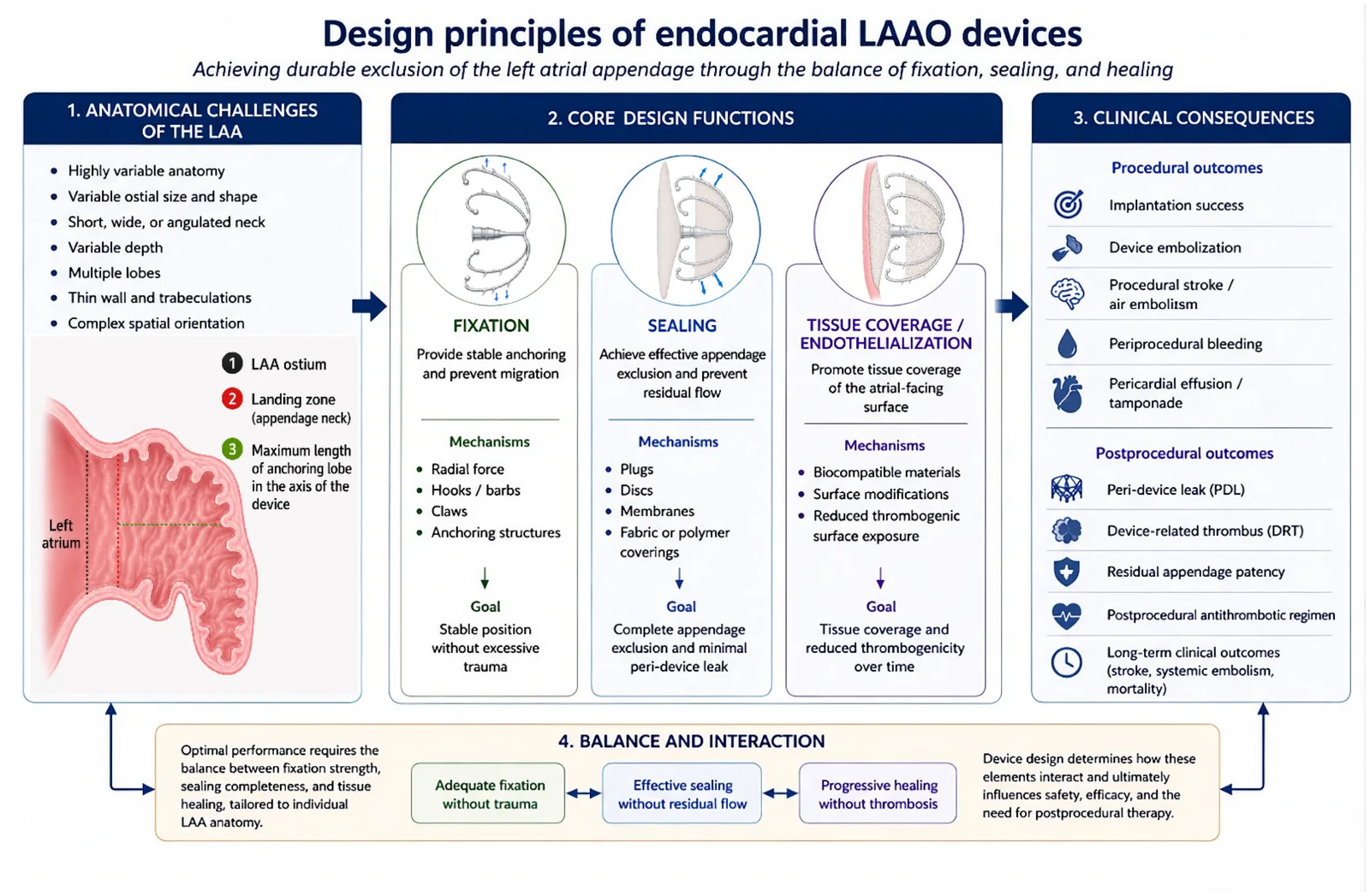
Conceptual framework of endocardial left atrial appendage occlusion (LAAO) device design. Endocardial LAAO success depends on the interaction between anatomical challenges, core device design functions—fixation, sealing, and tissue coverage/endothelialization—and clinical outcomes. Device design may influence procedural performance, residual appendage patency, peri-device leak, device-related thrombus, and the need for postprocedural antithrombotic therapy.

From a design perspective, successful LAAO depends on three interrelated functions: fixation, sealing, and tissue healing [8,9,12]. Fixation provides device stability and prevents migration or embolization; it may rely on radial force, hooks, barbs, claws, or other anchoring elements. Sealing determines the degree of residual communication between the left atrium and the appendage and is achieved through membranes, fabric coverings, discs, plugs, or other occlusive surfaces. Tissue healing, including progressive tissue coverage and endothelialization of the atrial-facing surface, is central to reducing thrombogenicity during the post-implant period and may influence the need for antithrombotic therapy after implantation [10,13]. These core design principles and their relationship to anatomical constraints and clinical outcomes are summarized in Figure 1.

These design functions must be balanced against the anatomical constraints of the LAA, which is thin-walled, trabeculated, and highly variable in ostial size, depth, angulation, and lobar structure [8,9,21]. Excessive radial force or traumatic anchoring may increase the risk of appendage injury, pericardial effusion, or tamponade, whereas insufficient fixation or incomplete sealing may predispose to device embolization, peri-device leak (PDL), or residual appendage patency [12,22,23]. Device-related thrombus (DRT) represents another clinically relevant consequence of the interaction between device surface, local flow conditions, tissue healing, and postprocedural antithrombotic strategy [10,13].

Based on structural configuration and occlusion strategy, endocardial LAAO devices can be broadly classified into plug-type devices, lobe-and-disc devices, umbrella-type or umbrella-disc devices, and newer alternative occlusion systems [8,9,24]. This classification is clinically relevant because each design category uses a different balance of fixation, sealing, and tissue-contact mechanisms, which may influence procedural complexity, anatomical suitability, residual leak patterns, device-related thrombus, and postprocedural management [8,9,10,11,12,13].

#### 3.2.2. Classification of Endocardial LAAO Devices

**Figure 2:**
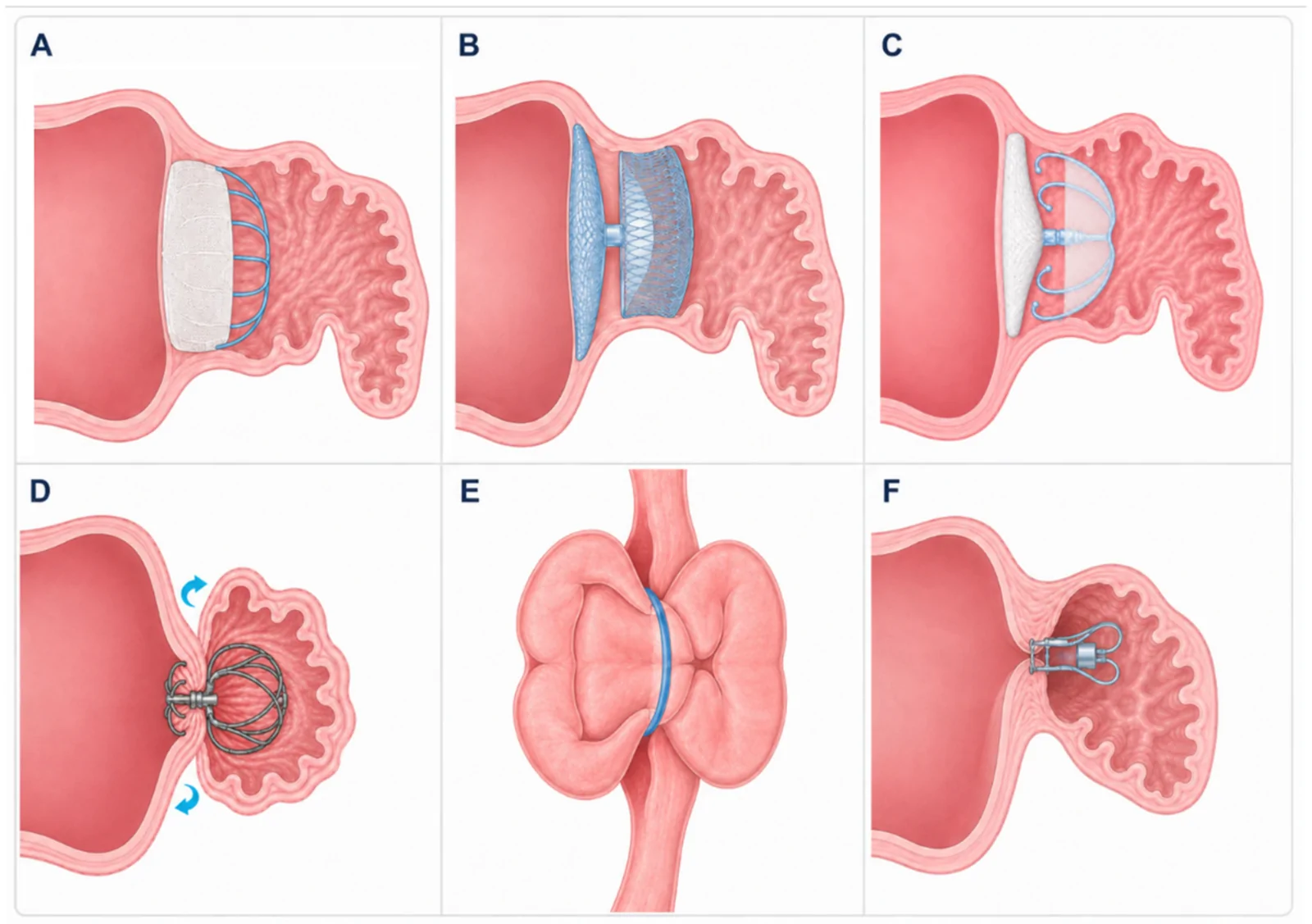
Conceptual design-based classification of endocardial left atrial appendage occlusion devices. Panel (**A**) illustrates a plug-type device, in which a single intraluminal plug seals the appendage at one sealing plane. Panel (**B**) shows a lobe-and-disc device, in which an intraluminal lobe is connected to an atrial-facing disc, providing a dual-seal mechanism. Panel (**C**) illustrates an umbrella-disc device, in which distributed umbrella-like anchoring elements engage the trabeculated appendage and are combined with disc sealing. Panels (**D**–**F**) show alternative occlusion concepts based on tissue twisting (blue arrow), inversion and compression, without a conventional intracavitary occluder. The figure illustrates simplified architectural principles rather than exact commercial device geometry.

The following sections summarize the major categories of endocardial LAAO devices, focusing on their mechanism of appendage exclusion, anatomical suitability, and current evidence base. Because many contemporary and investigational systems combine elements of more than one design concept, this classification is based on the dominant mechanism of appendage exclusion and fixation rather than minor device-specific variations. The main design categories are summarized in Table 3 and illustrated schematically in Figure 2, which emphasizes the architectural differences between plug-type, lobe-and-disc, umbrella-disc, and alternative occlusion concepts.

##### Plug-Type Devices

Plug-type devices are designed to occlude the LAA by positioning a self-expanding frame within the appendage and achieving sealing primarily through radial expansion against the landing zone [8,9,25]. Unlike disc-based systems, these devices generally avoid an external atrial disc and rely on a single intraluminal occlusion component, with fixation provided by radial force and circumferential anchors or barbs [12,25]. This compact architecture may simplify implantation and reduce the amount of device material exposed on the left atrial side, but it also makes adequate sizing, coaxial alignment, and circumferential apposition critical for durable sealing [9,12,25].

From a design perspective, plug-type devices may be well suited for anatomies with sufficient depth and a landing zone that allows stable intraluminal fixation [9,25]. However, reliance on a single sealing plane may be less forgiving in large, oval, shallow, angulated, or multilobulated appendages [22,25]. In such settings, incomplete apposition may predispose to residual peri-device leak or residual appendage patency, emphasizing the importance of preprocedural imaging, device sizing, and careful assessment of anatomical suitability [11,22].

Most plug-type devices consist of nitinol frames combined with fabric or polymer membranes that support flow exclusion and tissue coverage [8,9,25]. Newer-generation systems have focused on improving conformability, recapturability, visualization, and surface biocompatibility, aiming to reduce residual leak, device-related thrombus, and procedure-related complications [10,12,13,25].

The WATCHMAN platform remains the most established plug-type LAAO system, supported by randomized trials, prospective studies, and large real-world registries [15,16,26,27]. WATCHMAN FLX Pro represents a more recent iteration incorporating radiopaque markers, an expanded size range including a 40-mm device, and a HEMOCOAT surface modification designed to reduce device thrombogenicity [28]. Other plug-type or plug-like systems, including WaveCrest, Occlutech, CLAAS, Zenith, MemoLefort, and Lesifter, have more limited clinical evidence, and several remain investigational or supported mainly by early clinical or device-specific reports [8,9,24,29].

##### Lobe-and-Disc Devices

Lobe-and-disc devices use a dual-component architecture, combining an intraluminal lobe for fixation with an atrial-facing disc that covers the LAA ostium [8,9,30]. This design separates anchoring from sealing: the lobe provides stability within the appendage, whereas the disc compensates for irregular ostial geometry and provides an additional sealing surface [30,31]. Compared with plug-type devices, this architecture may be more adaptable to shallow, eccentric, or anatomically complex LAAs, particularly when circumferential apposition of a single plug is difficult to achieve [8,9,30].

The principal advantage of lobe-and-disc systems is the dual-seal concept, which may reduce conventional peri-device leak when the lobe and disc are appropriately sized and aligned [11,17,31,32]. However, residual patency after LAAO is device-specific and should not be interpreted solely as conventional peri-device leak [11,32]. Comparative imaging data from SWISS-APERO showed different residual patency patterns between Amulet and Watchman devices, including a higher rate of intradevice leak with Amulet and a higher rate of peri-device leak with Watchman, while overall LAA patency and short-term clinical outcomes were broadly similar [11,32]. These findings highlight the need for device-specific imaging interpretation after implantation.

The broader atrial-facing disc and more complex device geometry may also influence procedural handling and safety [30,31]. In comparative studies, Amulet implantation has been associated with higher rates of procedure-related complications, including pericardial effusion, compared with Watchman, although longer-term clinical outcomes have generally been comparable in randomized follow-up [17,31,33]. Therefore, improved ostial coverage should not be interpreted as uniformly superior procedural safety; device selection should remain individualized according to LAA anatomy, operator experience, and patient risk profile.

The Amplatzer Amulet is the principal established lobe-and-disc device and is both FDA-approved and CE-marked [30,31]. It is supported by randomized comparison with Watchman and longer-term follow-up data [17,30]. A next-generation Amulet platform, referred to as Amulet 360 in recent reports and as Amulet 2 in the VERITAS trial registration, is currently under clinical evaluation and aims to further improve conformability and sealing performance [34]. Other lobe-and-disc systems include Ultraseal, LACbes, Omega, and SeaLA/e-SeaLA; however, available evidence for these platforms remains limited, and several are supported mainly by early clinical experience, registries, or investigational data [8,24,35,36].

##### Umbrella-Type Devices

Umbrella-type or umbrella-disc devices use expandable anchoring frameworks designed to engage the trabeculated LAA wall. In contrast to plug-type devices, which rely primarily on circumferential apposition of an intraluminal frame, and lobe-and-disc devices, which use a lobe for fixation and a disc for ostial coverage, umbrella-type systems typically incorporate multiple claws, hooks, or umbrella-like anchoring elements combined with a sealing disc [8,9,12]. This configuration may be advantageous in complex, multilobulated, shallow, or irregular LAAs where conventional plug- or lobe-based anchoring may be less stable [8,9,24].

The potential advantage of this design is its ability to achieve stability by engaging the trabeculated appendage rather than relying exclusively on a single circumferential landing zone [8,9,24]. This may improve conformability and anchoring in anatomically challenging appendages. However, increased device–tissue interaction and multiple anchoring elements may also introduce specific safety considerations, including appendage trauma, pericardial effusion, residual leak, or device-related thrombus [12]. Compared with Watchman and Amulet platforms, the clinical implications of these design features remain less well defined because robust randomized and long-term comparative data are limited [8,9,24,37,38].

The LAmbre device is the most established representative of this category. It consists of an umbrella-like anchoring component and an atrial-facing sealing disc, with the possibility of selecting different lobe and disc sizes to accommodate variable ostial and appendage anatomies [37,38]. LAmbre received CE marking in 2016 and has been evaluated mainly in observational studies and early clinical experience, with available data suggesting procedural feasibility and acceptable short- to mid-term outcomes [37,38]. Nevertheless, evidence remains less mature than for the FDA-approved Watchman and Amulet platforms, and direct comparisons with contemporary-generation devices are limited.

Ongoing randomized evaluation of LAmbre Plus may better define its comparative safety and efficacy against commercially available transcatheter LAAO devices [39]. Other investigational umbrella-like systems, including LAmax and Leftear, remain at earlier stages of clinical evaluation [8,9,24,40]. Their future role will depend on whether they can demonstrate durable appendage exclusion, low rates of peri-device leak and device-related thrombus, and acceptable procedural safety across complex LAA anatomies.

##### Novel and Alternative Occlusion Concepts

Beyond established plug-type, lobe-and-disc, and umbrella-disc architectures, several emerging LAAO technologies aim to address persistent limitations of conventional devices, including incomplete sealing, device-related thrombus, complex appendage anatomy, and the need for prolonged postprocedural antithrombotic therapy [8,9,10,11,12,13,24]. These innovations can be broadly divided into two groups: incremental design refinements of existing endocardial occluder concepts and alternative strategies that modify or exclude the appendage through non-conventional mechanisms [9,24,41,42].

Incremental design refinements focus on improving conformability, reducing thrombogenic surface exposure, facilitating implantation, and enhancing adaptation to complex LAA anatomy [8,9,12,24]. Surface modifications represent one such strategy. WATCHMAN FLX Pro incorporates the HEMOCOAT surface modification, whereas LAmax has been described with a membrane modification approach intended to reduce thrombogenicity and promote biocompatibility [28,40]. Other systems emphasize structural adaptability. Ultraseal incorporates an articulating lobe and radiopaque markers to facilitate positioning, while devices such as Amulet 360/Amulet 2, Omega, and LACbes aim to improve conformability through modifications in lobe–disc geometry, waist flexibility, or sealing surface design [24,34].

The CLAAS system represents a distinct plug-like approach, using a highly conformable foam structure supported by a nitinol endoskeleton. By accommodating a broad range of appendage dimensions with a limited number of device sizes, this design is intended to simplify preprocedural sizing and reduce dependence on exact landing-zone measurements [43]. Other device-specific innovations include separate selection of lobe and disc sizes, as used in LAmbre, and modified disc geometries intended to improve ostial coverage in irregular appendages [24,44]. However, for many of these systems, clinical evidence remains limited to early feasibility studies, registries, or investigational reports [8,9,24].

A separate group of technologies departs more substantially from conventional intracavitary occluder design. These systems aim to achieve LAA exclusion by mechanically modifying the appendage rather than simply implanting a plug, lobe-disc, or umbrella-disc device within it. Examples include tissue compression or twisting concepts such as Laminar, appendage inversion or ligation-like approaches such as Appligator, and tissue pinching or compression systems such as Endomatic [9,41,42]. These approaches may theoretically reduce long-term intracavitary metal burden or alter the healing response, but they remain at an early stage of clinical evaluation [9,24,41,42]. Their future role will depend on whether they can demonstrate durable appendage exclusion, low rates of residual patency and device-related thrombus, procedural safety, and meaningful advantages over established LAAO platforms.

#### 3.2.3. Overview of Contemporary and Emerging Devices

Representative endocardial LAAO devices are summarized in Table 4a,b. Table 4a presents currently certified and previously certified devices, including their design category, fixation strategy, radiographic visibility, available size range, and regulatory status. Table 4b summarizes emerging and investigational devices according to design type, fixation mechanism, reported size range, and current stage of clinical development and supporting evidence.

### 3.3. Anatomical and Imaging Considerations for Device Selection

**Figure 3:**
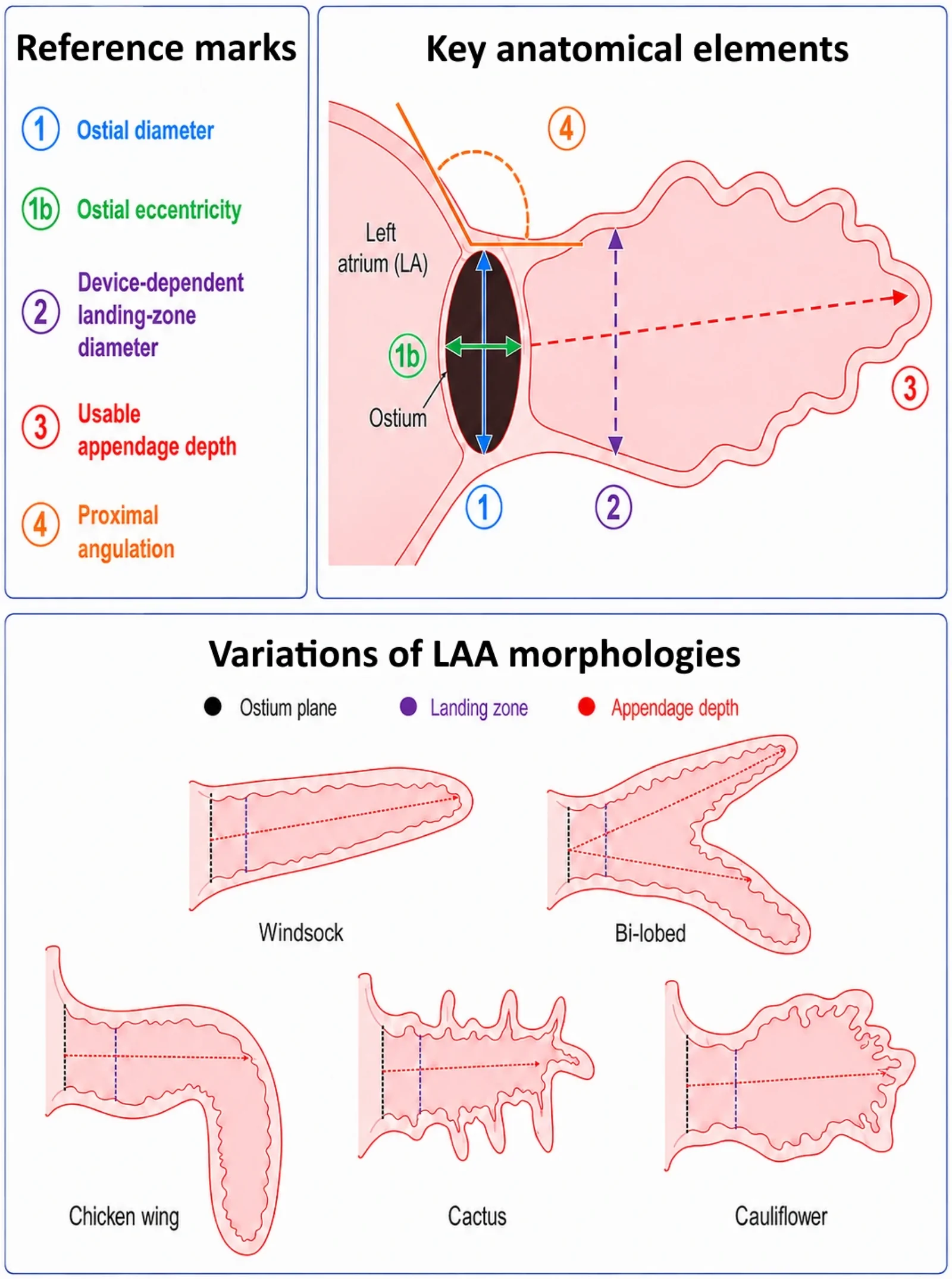
Key anatomical parameters for endocardial LAAO planning. The schematic illustrates anatomical landmarks relevant to device sizing and selection, including ostial dimensions, device-dependent landing-zone diameter, usable appendage depth, proximal angulation, lobar anatomy, and trabeculation. Measurements are shown conceptually; the exact landing-zone definition and sizing plane are device-specific and should be interpreted together with preprocedural and intraprocedural imaging findings.

The clinical relevance of LAAO device design becomes most apparent during anatomical assessment and procedural planning. The LAA is a thin-walled, trabeculated, and highly variable structure, with substantial interindividual differences in ostial size and shape, neck configuration, device-dependent landing-zone dimensions, usable appendage depth, proximal angulation, and lobar anatomy (Figure 3). These parameters influence device sizing, coaxial alignment, anchoring stability, sealing completeness, and the risk of residual patency after implantation [8,9,21].

Several morphological classifications have been proposed, most commonly categorizing the LAA as chicken wing, windsock, cactus, or cauliflower [45]. These categories are useful for anatomical description and procedural communication, and observational data have suggested that non-chicken-wing morphologies may be associated with a higher risk of stroke or transient ischemic attack than chicken-wing morphology [45]. However, for procedural planning, morphology alone is insufficient. Device selection depends more directly on measurable parameters, including ostial diameter and eccentricity, device-dependent landing-zone diameter, usable appendage depth, proximal angulation, number and orientation of lobes, and the presence of a short, broad, or sharply angulated neck (Figure 3). Accordingly, morphological categories should be interpreted within a broader imaging-based anatomical assessment rather than used as isolated determinants of device choice [21,22,45].

Preprocedural and intraprocedural imaging are central to translating LAA anatomy into device strategy. Before implantation, current recommendations support anatomical assessment with either TEE or cardiac CT, and in contemporary practice these modalities are often used complementarily [7,21]. CT provides high-resolution three-dimensional anatomical assessment and may improve sizing in complex or uncertain anatomies, whereas TEE remains important for thrombus exclusion, dynamic assessment, and peri- or intraprocedural decision-making [21,46,47]. Comparative data suggest that CT-based planning may reduce device size changes and procedural inefficiency, partly by improving assessment of maximal landing-zone dimensions that may be underestimated by two-dimensional TEE [47].

During implantation, imaging shifts from anatomical planning to procedural guidance. TEE remains the most established modality for guiding transseptal access, catheter orientation, device positioning, deployment, stability assessment, and immediate evaluation of residual flow or pericardial effusion [21]. ICE is increasingly used in selected centers as an intraprocedural alternative, particularly when avoidance of general anesthesia or transesophageal imaging is desirable [21].

From a device-selection perspective, different anatomical patterns may favor different design strategies, although anatomy-based device matching remains incompletely standardized [21]. Plug-type devices generally require sufficient appendage depth and a landing zone that allows stable circumferential apposition of the intraluminal frame [9,12,25]. They may be less forgiving in very shallow, markedly oval, sharply angulated, or multilobulated appendages, where incomplete apposition may predispose to peri-device leak or residual appendage patency [22,25,32]. Lobe-and-disc devices may be useful when ostial geometry is irregular or when additional ostial coverage is desirable, because the lobe provides intraluminal fixation while the disc provides a second sealing surface [11,17,31]. Umbrella-type systems may be conceptually attractive in complex or trabeculated appendages because distributed anchoring elements can engage the LAA wall beyond a single circumferential landing zone [8,9,21]. However, robust comparative evidence supporting anatomy-based selection of these devices remains limited.

Postprocedural imaging, typically with TEE and/or cardiac CT, is used to assess device position, exclude device-related thrombus, and determine whether residual appendage patency or peri-device leak is present. When residual flow is detected, its mechanism, location, and extent should be characterized, because these findings may influence the intensity and duration of antithrombotic therapy, the need for repeat imaging, and, in selected cases, consideration of further intervention [21,22,32]. Peri-device leak may result from undersizing, incomplete circumferential apposition, eccentric ostial geometry, device malalignment, complex lobar anatomy, or insufficient sealing surface [22]. Importantly, residual patency is not uniform across devices and should be interpreted in a device-specific manner. In SWISS-APERO, Amulet and Watchman showed different residual patency patterns, with higher rates of conventional peri-device leak after Watchman and higher rates of intradevice leak after Amulet in subsequent imaging analyses. Overall LAA patency and short-term clinical outcomes were broadly similar, underscoring that follow-up imaging should define the mechanism and location of residual patency rather than simply classify residual flow as present or absent [11,32].

Taken together, anatomical assessment and multimodality imaging provide the practical link between device design, procedural planning, and postprocedural outcomes. Device selection should integrate measurable anatomical parameters, expected fixation and sealing behavior, and the anticipated risk of residual patency or device-related thrombus [21,22,32,46]. Key anatomical features relevant to device selection and procedural planning are summarized in Table 5.

### 3.4. Clinical Evidence and Comparative Effectiveness

The clinical evidence supporting endocardial LAAO has evolved in parallel with changes in anticoagulation practice and device technology. Early randomized trials were conducted in the vitamin K antagonist era and primarily evaluated the WATCHMAN device against warfarin, whereas contemporary studies increasingly assess newer-generation devices, direct device-to-device comparisons, and the comparative effectiveness of LAAO against direct oral anticoagulant (DOAC)-based or physician-directed medical therapy [14,15,16,18,19].

The first major randomized evidence came from the PROTECT AF and PREVAIL trials, which compared WATCHMAN implantation with warfarin therapy in patients with non-valvular AF [15,16]. These trials established LAAO as a non-inferior alternative to warfarin-based stroke prevention in selected anticoagulation-eligible patients, while also highlighting the importance of procedural safety and operator experience. Longer-term pooled follow-up suggested comparable stroke prevention with reductions in hemorrhagic stroke, major bleeding, and mortality after LAAO, although these findings should be interpreted in the context of warfarin-era management and first-generation device experience [48].

Contemporary WATCHMAN evidence has focused on newer-generation platforms and real-world implementation. The PINNACLE FLX study evaluated WATCHMAN FLX and supported high implant success and favorable short-term safety with a next-generation device design [27]. Large registry data have extended these findings into routine clinical practice, demonstrating broad adoption and generally favorable procedural outcomes, while also showing that real-world outcomes depend on patient risk profile, comorbidity burden, and procedural experience [26,49].

The Amplatzer Amulet has the most robust comparative evidence among lobe-and-disc devices. In the Amulet IDE trial, Amulet was compared with WATCHMAN 2.5 and demonstrated non-inferior safety and effectiveness, with superior LAA occlusion rates but a different procedural and device-related profile [31]. Five-year follow-up demonstrated sustained safety and effectiveness of Amulet and supports the dual-seal platform as a clinically established alternative to WATCHMAN, although the comparator was WATCHMAN 2.5 rather than WATCHMAN FLX or FLX Pro [17].

Device-to-device studies also illustrate why anatomical and imaging outcomes should not be reduced to a binary concept of “sealed” versus “not sealed.” SWISS-APERO compared Amulet with WATCHMAN FLX and found no superiority of Amulet for CT-assessed LAA patency at 45 days [11]. Subsequent imaging analyses demonstrated device-specific residual patency patterns, supporting the need for device-specific interpretation of follow-up imaging findings [32].

The introduction of DOACs changed the central comparative question from whether LAAO could replace warfarin to whether it can provide a favorable alternative to contemporary anticoagulation. PRAGUE-17 compared LAAO with DOAC therapy in high-risk patients and demonstrated non-inferiority for a composite of cardiovascular, neurological, and bleeding events [50]. Longer-term follow-up confirmed non-inferiority and showed fewer nonprocedural bleeding events after LAAO [14].

More recent randomized studies have expanded this question into distinct clinical populations. OPTION evaluated LAAO after catheter ablation in patients at elevated stroke risk and reported less nonprocedural major or clinically relevant nonmajor bleeding compared with oral anticoagulation, while maintaining non-inferiority for major efficacy outcomes [51]. CHAMPION-AF evaluated LAAO as a first-line alternative to non-vitamin K antagonist oral anticoagulants in anticoagulation-eligible patients; the trial met non-inferiority criteria for the primary efficacy endpoint and showed lower nonprocedural bleeding, but interpretation requires caution because of low event rates, non-inferiority margin considerations, and a numerical excess of ischemic stroke in the LAAO group [18]. In contrast, CLOSURE-AF tested LAAO against physician-directed best medical therapy in an older, high-risk population with elevated thromboembolic and bleeding risk; LAAO did not meet non-inferiority for the composite endpoint of stroke, systemic embolism, major bleeding, or cardiovascular or unexplained death [19].

Taken together, current evidence supports LAAO as a selective alternative to long-term anticoagulation rather than a universal replacement. The strongest clinical evidence remains concentrated around WATCHMAN and Amulet platforms, whereas evidence for many emerging devices remains limited to early clinical experience, registries, or ongoing trials. Contemporary data increasingly suggest that the net benefit of LAAO depends on the interaction between patient selection, procedural risk, device design, residual patency, and postprocedural antithrombotic strategy. Major randomized and comparative studies are summarized in Table 6.

Ongoing LAAO research is moving beyond proof of efficacy toward next-generation devices, combined ablation–closure strategies, simplified procedural guidance, optimized post-implant antithrombotic regimens, and broader evaluation of LAAO as both an alternative and an adjunct to oral anticoagulation. A summary of these trials is presented in Table 7.

### 3.5. Postprocedural Antithrombotic Management

Following LAAO, antithrombotic therapy is required during the early post-implant period, when the device surface remains exposed to circulating blood and before complete tissue coverage has occurred. The central challenge is to prevent DRT while preserving the anticipated reduction in bleeding risk that underlies the rationale for LAAO in many patients. Consequently, postprocedural therapy should be individualized according to thromboembolic risk, bleeding risk, device type, procedural result, and follow-up imaging findings.

Contemporary guidelines do not establish a single universal postimplant antithrombotic regimen after LAAO. The 2025 SCAI/HRS guideline suggests either OAC or DAPT following LAAO (conditional recommendation, low-certainty evidence), with DAPT considered particularly reasonable in patients with significant contraindications to OAC, such as severe bleeding history or risk [7]. No recommendation is made regarding SAPT versus other antithrombotic regimens because of insufficient evidence, and the optimal duration of OAC or DAPT remains uncertain. The 2024 ESC atrial fibrillation guideline similarly emphasizes the heterogeneity of contemporary post-LAAO antithrombotic practice, noting that DOAC-based and antiplatelet-only approaches have been used as alternatives to historical VKA-based protocols and that, in the absence of robust randomized evidence, treatment decisions are generally individualized [1]. Accordingly, trial-tested protocols should be distinguished from device-labelled regimens, observational practice, and emerging strategies outside established device-labelled pathways, as these categories differ in the nature and strength of their supporting evidence.

Historically, postprocedural antithrombotic management after LAAO was largely derived from the pivotal WATCHMAN trial protocols. In PROTECT AF and PREVAIL, patients received VKA plus aspirin for 45 days, followed by DAPT (aspirin plus clopidogrel) for 6 months and lifelong aspirin, provided follow-up imaging excluded device-related thrombus and clinically significant peri-device leak [15,16]. This regimen became the reference strategy for early WATCHMAN use, but its applicability to contemporary practice is limited because many patients referred for LAAO have prior bleeding or contraindications to anticoagulation.

This mismatch has driven evaluation of less anticoagulant-intensive strategies. Observational studies in OAC-ineligible patients suggest that DAPT-based regimens after WATCHMAN implantation may be feasible in selected patients, although evidence remains limited by nonrandomized design and heterogeneous protocols [52,53]. In Amulet IDE, DAPT was commonly used after Amulet implantation and was associated with outcomes comparable to the VKA-based regimen used in the WATCHMAN arm [17,31]. PRAGUE-17 further illustrates a risk-adapted postprocedural approach, using antithrombotic therapy tailored to bleeding and thrombotic risk after LAAO in a DOAC-comparator randomized trial [14]. However, these regimens reflect specific study protocols and should not be interpreted as universal post-LAAO treatment algorithms. Evidence-derived postprocedural antithrombotic strategies after LAAO are summarized in Table 8.

Randomized evidence for DOAC-based post-LAAO regimens remains limited but is evolving. ADRIFT provided early randomized proof-of-concept evidence supporting reduced-dose rivaroxaban over DAPT in reducing coagulation activation after LAAO; however, it was a small trial with a biomarker primary endpoint and was not designed to support conclusions regarding clinical outcomes [56]. Data from the ADALA randomized clinical trial, comparing low-dose DOAC with DAPT after LAAO, showed reduced device-related thrombus and improved safety signals in the DOAC group, although the study included only 90 participants and was terminated early [54]. More recently, ANDES compared short-term DOAC therapy with DAPT after LAAO and suggested comparable efficacy with a potential safety advantage for DOAC-based therapy, although further confirmation is needed [55]. Taken together, these trials support the biological and clinical plausibility of short-term or low-dose DOAC therapy after LAAO, but they remain insufficient to establish a universal DOAC-based standard.

Meta-analyses provide a broader, but less definitive, perspective. Overall, anticoagulant-based regimens, particularly DOAC-based strategies, appear to be associated with lower risks of thromboembolism and device-related thrombus than antiplatelet-only therapy, whereas single antiplatelet therapy or no therapy may be associated with higher adverse event rates in some analyses [57,58]. However, heterogeneity in devices, patient risk profiles, treatment regimens, and imaging surveillance limits the clinical interpretability of these analyses, which remain hypothesis-generating rather than practice-defining.

Several ongoing studies are expected to clarify whether post-LAAO therapy can be safely de-escalated or individualized. SIMPLAAFY is evaluating aspirin alone, reduced-dose DOAC followed by aspirin, and DAPT after WATCHMAN FLX Pro implantation [59]. FADE-DRT is comparing standard DAPT, genotype-guided antithrombotic therapy, and low-dose DOAC therapy in patients undergoing LAAO with contraindications to OAC [60]. SAFE-LAAC is evaluating shorter versus longer DAPT duration, followed by single antiplatelet therapy or antithrombotic discontinuation [61]. These studies may help define lower-intensity strategies for selected patients, but they will also need to clarify which factors should guide regimen selection, including bleeding risk, thromboembolic risk, device type, procedural result, and follow-up imaging findings. A summary of these trials is presented in Table 7.

Overall, postprocedural antithrombotic management after LAAO is evolving from fixed trial-derived protocols toward individualized, risk-adapted strategies. Future algorithms will need to define not only the relative role of DOAC- versus antiplatelet-based therapy, but also how treatment intensity and duration should be modulated by patient risk profile, device design, residual patency, and imaging-defined tissue coverage.

## 4. Discussion

The present review highlights the evolution of endocardial LAAO from a relatively limited set of mechanical occluders into a heterogeneous field of device platforms with distinct anchoring, sealing, and healing characteristics. Although LAAO remains primarily positioned as a stroke-prevention strategy for selected patients with AF who are unsuitable for, or poorly tolerate, long-term oral anticoagulation, contemporary practice increasingly requires integration of patient risk, appendage anatomy, device design, procedural result, and postprocedural antithrombotic management.

A key message is that LAAO devices should not be regarded as interchangeable mechanical barriers. Plug-type, lobe-and-disc, umbrella-disc, and alternative occlusion systems differ in how they achieve fixation, sealing, and tissue contact [8,9,12]. These differences may influence anatomical suitability, residual patency, device-related thrombus, and procedural safety [11,12,13,32]. However, evidence supporting formal anatomy-based device selection remains limited, and most current decision-making still relies on imaging interpretation, operator experience, and device availability rather than validated comparative algorithms [8,9,21].

The clinical evidence base remains strongest for the WATCHMAN and Amulet platforms. Warfarin-era trials established LAAO as a non-inferior alternative to VKA therapy in selected anticoagulation-eligible patients [15,16], but the introduction of DOACs substantially changed the comparative landscape. Contemporary trials suggest that LAAO may provide comparable efficacy and reduce long-term nonprocedural bleeding in selected populations, yet findings from PRAGUE-17, OPTION, CHAMPION-AF, and CLOSURE-AF also show that net clinical benefit depends strongly on patient selection, procedural risk, comorbidity burden, and competing bleeding and thromboembolic risks [14,18,19,51].

CHAMPION-AF evaluated WATCHMAN FLX as a first-line alternative to NOAC therapy in anticoagulation-eligible patients [18]. At 3 years, LAAO was non-inferior for cardiovascular death, stroke, or systemic embolism (5.7% vs. 4.8%; difference 0.9 percentage points, 95% CI −0.8 to 2.6; *p* < 0.001 for non-inferiority; NI margin 4.8 percentage points) and reduced nonprocedural bleeding (10.9% vs. 19.0%; HR 0.55, 95% CI 0.45–0.67; *p* < 0.001 for superiority). However, interpretation requires caution because event rates were lower than anticipated and ischemic stroke or systemic embolism was numerically higher with LAAO (3.2% vs. 2.2%). The prespecified 5-year analysis of this endpoint is ongoing.

CLOSURE-AF evaluated LAAO versus physician-directed best medical care in patients with both high thromboembolic and bleeding risk [19]. LAAO did not meet non-inferiority for the composite of stroke, systemic embolism, major bleeding, or cardiovascular or unexplained death (16.8 vs. 13.3 events/100 patient-years; *p* = 0.44 for non-inferiority; NI margin HR 1.30). However, stroke or systemic embolism rates were nearly identical (2.9 vs. 2.8 events/100 patient-years), whereas major bleeding (7.4 vs. 6.2 events/100 patient-years) and cardiovascular or unexplained death (9.5 vs. 7.7 events/100 patient-years) were numerically higher with LAAO. Periprocedural complications occurred in 5.7% of patients. Thus, failure to demonstrate non-inferiority appears to reflect the overall balance of procedural risk, bleeding, and competing mortality rather than a clear difference in stroke prevention alone.

The apparently different findings of CHAMPION-AF and CLOSURE-AF should also be interpreted in the context of markedly different study populations. CHAMPION-AF enrolled a younger and lower-risk anticoagulation-eligible population (mean age 71.7 years, CHA_2_DS_2_-VASc 3.5, HAS-BLED 1.3), whereas CLOSURE-AF included substantially older and higher-risk patients (mean age 77.9 years, CHA_2_DS_2_-VASc 5.2, HAS-BLED 3.0), including 30.4% with previous BARC 3a/3b major bleeding and 24.1% with stage IV chronic kidney disease [18,19]. These differences suggest that comorbidity burden, bleeding risk, and procedural vulnerability may substantially influence the net clinical benefit of LAAO and help explain the divergent overall trial results.

Postprocedural antithrombotic therapy remains a major unresolved issue after LAAO. Traditional regimens were derived from early WATCHMAN trial protocols, whereas contemporary practice increasingly includes DAPT-based, reduced-dose DOAC, and individualized approaches [10,15,16]. Emerging randomized data suggest that short-term or low-dose DOAC strategies may be promising in selected patients, but evidence remains insufficient to define a universal regimen [54,55,56]. Importantly, device design may influence residual patency, device-related thrombus, and tissue coverage, yet postprocedural therapy is still largely determined by patient bleeding and thrombotic risk rather than by validated device-specific algorithms [10,11,13].

The expanding number of investigational devices reflects ongoing efforts to improve conformability, reduce thrombogenic surface exposure, simplify sizing, and treat more complex anatomies [9,24,40,43,62,63,64]. However, for many emerging systems, evidence remains limited to early feasibility studies, registries, or preclinical data [9,24,38,43,62,63,64]. Without adequately powered comparative studies, it remains uncertain whether novel design features will translate into clinically meaningful improvements over established platforms.

Overall, current evidence supports a shift from a device-centered approach toward an integrated patient–anatomy–device–therapy model of LAAO. Until more robust comparative data are available, LAAO should be applied selectively, with careful integration of clinical risk, anatomical suitability, procedural expertise, device characteristics, follow-up imaging findings, and postprocedural antithrombotic management. Future studies should determine whether systematic imaging-based device selection, standardized assessment of residual patency and device-related thrombus, and individualized antithrombotic regimens can improve outcomes across different LAAO platforms.

## 5. Conclusions

Endocardial LAAO has evolved from a mechanical alternative to oral anticoagulation into a heterogeneous and increasingly individualized field of device-based stroke prevention. Contemporary devices differ substantially in their anchoring, sealing, surface characteristics, and interaction with LAA anatomy, and these differences may influence procedural success, residual patency, device-related thrombus, and postprocedural management.

Current evidence supports LAAO as a selective strategy for carefully chosen patients with AF, particularly when long-term anticoagulation is contraindicated, poorly tolerated, or associated with an unfavorable risk–benefit profile. However, LAAO should not be regarded as a universal replacement for anticoagulation. The strongest clinical evidence remains concentrated around the WATCHMAN and Amulet platforms, whereas data for many emerging devices remain limited.

Future progress will require a more integrated approach linking patient selection, imaging-based anatomical assessment, device-specific design features, procedural outcomes, and individualized antithrombotic therapy. Until more robust comparative data are available, LAAO should be applied through careful shared decision-making and tailored to the interaction between patient risk, appendage anatomy, device characteristics, and postprocedural imaging findings.

## Figures and Tables

**Table 1:** Clinical scenarios that may complicate or limit long-term oral anticoagulation in patients with atrial fibrillation [1,5,7,20].

Clinical Condition	Representative Examples
High bleeding risk	Advanced age or frailty; chronic kidney disease; liver disease; anemia or thrombocytopenia; concomitant antiplatelet or NSAID use
Prior major bleeding	Previous intracranial hemorrhage; gastrointestinal bleeding; major bleeding requiring hospitalization or transfusion; recurrent clinically significant bleeding
Non-reversible contraindication or intolerance to long-term OAC	Non-reversible bleeding predisposition; recurrent major bleeding despite correction of modifiable factors; clinically significant intolerance to an appropriate anticoagulant regimen
Difficulty maintaining appropriate long-term anticoagulation	Labile INR despite appropriate monitoring; clinically important drug–drug or drug–food interactions affecting anticoagulation; inability to maintain an appropriate anticoagulation regimen despite reasonable attempts at optimization

NSAIDs—non-steroidal anti-inflammatory drugs; INR—international normalized ratio.

**Table 2:** Guideline positioning of LAAO in atrial fibrillation [1,5,7].

Guideline	Recommendation	Clinical Interpretation
ACC/AHA/ACCP/HRS 2023	**Class IIa** in AF with moderate-to-high stroke risk and a nonreversible contraindication to long-term OAC; **Class IIb** in AF with high bleeding risk on OAC	Provides the strongest recommendation among current major guidelines; distinguishes between true contraindication and elevated bleeding risk
ESC 2024	**Class IIb** in AF with contraindications to long-term anticoagulation	More conservative positioning; emphasizes correction of modifiable bleeding risk factors
SCAI/HRS 2025	**Conditional recommendation** for selected patients with non-valvular AF not suitable for long-term OAC	Highlights shared decision-making and individualized assessment of bleeding and thromboembolic risk

ACC—American College of Cardiology; AHA—American Heart Association; ACCP—American College of Chest Physicians; HRS—Heart Rhythm Society; ESC—European Society of Cardiology; SCAI—Society for Cardiovascular Angiography & Interventions; AF—atrial fibrillation; LAAO—left atrial appendage occlusion; OAC—oral anticoagulation.

**Table 3:** Design-based classification of endocardial left atrial appendage occlusion devices.

Device Design Category	Dominant Mechanism	Main Design Strength	Main Limitation/Trade-Off	Representative Examples
Plug-type devices	Intraluminal plug positioned within the LAA; sealing by radial expansion against the landing zone; fixation by radial force ± anchors/barbs	No external atrial disc; strongest clinical evidence for WATCHMAN platform	Relies on circumferential forces at a single sealing plane; may be less forgiving in shallow, oval, angulated, or multilobulated LAAs	WATCHMAN, WATCHMAN FLX/FLX Pro, Occlutech, WaveCrest, CLAAS
Lobe-and-disc devices	Intraluminal lobe provides fixation; atrial-facing disc covers the ostium; dual-seal concept	Better ostial coverage; potentially useful in irregular ostia or shallow landing zones; more adaptable to variable anatomy	More left atrial-facing material; more complex geometry; device-specific residual patency patterns, including intradevice leak	Amplatzer Amulet, Amulet 360/Amulet 2, Ultraseal, LACbes, Omega, SeaLA/e-SeaLA
Umbrella-type devices	Umbrella-like expandable anchoring framework engages trabeculated LAA wall; often combined with disc sealing	Potentially useful in complex, multilobulated, shallow, or irregular appendages	More anchoring elements and device–tissue interaction; limited randomized comparative evidence versus WATCHMAN/Amulet	LAmbre, LAmbre Plus, LAmax, Leftear
Novel/alternative occlusion concepts	Non-conventional appendage exclusion: tissue compression, inversion, ligation-like mechanisms, absorbable or polymer-based systems	May overcome limitations of conventional plug/disc systems; may reduce long-term metal burden or change healing profile	Mostly early-stage, investigational, preclinical, or limited clinical data; uncertain durability and comparative safety	Laminar, Appligator, Endomatic, bioabsorbable/hydrogel/polymer-based concepts

Devices are grouped according to the dominant mechanism of appendage exclusion and fixation. Some contemporary and investigational systems combine features of more than one category; therefore, this classification should be interpreted as a pragmatic framework rather than a rigid taxonomy.

**Table 4:** (**a**). Overview of representative currently certified and previously certified endocardial left atrial appendage occlusion devices. (**b**). Evidence and development status of representative emerging endocardial left atrial appendage occlusion devices.

(**a**)					
**Device (Manufacturer)**	**Type**	**Fixation Mechanism**	**Radiopaque Markers**	**Size Range (mm)**	**Regulatory Status**
WATCHMAN FLX Pro (Boston Scientific, Marlborough, MA, USA)	Plug	Two rows of fixation anchors + radial force	Yes; 3 markers	20–40	FDA-approved; CE-marked; commercially available
Amplatzer Amulet (Abbott Medical, Plymouth, MN, USA)	Lobe–disc	Distal lobe stabilization wires/anchors + proximal disc sealing	NR	16–34	FDA-approved; CE-marked; commercially available internationally
LAmbre (LifeTech Scientific, Shenzhen, China)	Umbrella–disc	Distal umbrella/claws + proximal cover	NR	16–36; multiple configurations	CE-marked; NMPA approved; commercially available in multiple markets
Ultraseal II (Cardia, Inc., Eagan, MN, USA)	Lobe–disc/bulb–sail	Distal fixation hooks + articulating joint	Yes; distal radiopaque markers	16–34	CE-marked; commercial availability is uncertain
MemoLefort (Lepu ScienTech Medical Technology, Shanghai, China)	Plug	Barbed anchoring system	NR	21–33	NMPA approved in China; commercially marketed in China
SeaLA (Hangzhou Valued Medtech, Hangzhou, China)	Lobe–disc	Anchoring disc with barbs + sealing disc	NR	~16–36	NMPA approved in China
LACbes (Shanghai Push Medical Devices, Shanghai, China)	Lobe–disc	Curved/isogenous barbs	NR	16–34, 2-mm increments	NMPA approved; commercially available in China
LAMax (Shenzhen KYD Biomedical Technology, Shenzhen, China)	Umbrella–disc/anchor–disc	Embedded hooks/barbs	NR	NR	NMPA approved in China
Leftear/LeftEAR (Guangdong Pulse Medical Technology, Zhuhai, China)	Dual-seal, double-body inner-plug	Fixation disc + occlusion disc	NR	NR	NMPA approved in China
Occlutech LAA Occluder—original generation (Occlutech International, Helsingborg, Sweden)	Plug	Distal loop/rim fixation	NR	18–33	Previously CE-marked; subsequently recalled/withdrawn because of device dislodgement/embolization concerns
WaveCrest (Biosense Webster, Irvine, CA, USA)	Plug	Retractable fixation anchors	NR	22–32	Historically CE-marked and clinically investigated; commercial availability is uncertain
OMEGA (Eclipse Medical, Dublin, Ireland)	Lobe–disc/cup–disc	Fixation hooks + radial lobe	Platinum-coated implant; separate marker status NR	14–30	Previously commercially distributed; worldwide recall in November 2024; commercial availability is uncertain
(**b**)					
**Device (Manufacturer)**	**Type**	**Fixation Mechanism**	**Size Range (mm)**	**Current Development Status**	
CLAAS AcuFORM (Conformal Medical, Nashua, NH, USA)	Conformable foam plug	Two rows of low-profile anchors	Two-size platform	Pivotal IDE evaluation; CONFORM randomized pivotal trial (NCT05147792) actively recruiting; not commercially available	
Laminar LAAX (Biosense Webster, Irvine, CA, USA)	Tissue-elimination/ball-and-lock system	Tissue compression rather than conventional anchors	12 and 16 reported	Pivotal IDE stage; randomized pivotal trial (NCT06168942) currently on hold; investigational	
LeSifter (Nanjing YDB Medical Technology, Nanjing, China)	Plug	Circumferential/barbed fixation	20–35 reported	Multicenter clinical evaluation completed; 5-year prospective follow-up ongoing (ChiCTR2000030590); regulatory approval not independently verified	
Zenith (AuriGen Medical, Galway, Ireland)	Plug	Independent anchors	NR	Early clinical feasibility stage; first-in-human study (NCT05951101) ongoing/expanding; no pivotal evaluation yet	
eSeaLA (Hangzhou Dinova EP Technology, Hangzhou, China)	Combined LAAO–PFA system	Mechanical anchoring combined with pulsed-field ablation	NR	First-in-human feasibility stage (NCT05731882); combined PFA–LAAO system; no pivotal evaluation or marketing approval reported	
SEPIOLA (Endomatic, Or Yehuda, Israel)	Mechanical compression/closure system	Mechanical tissue compression	N/A/NR	Early clinical feasibility stage; safety/performance study (NCT06099106) recruiting; no pivotal evaluation yet	
Append System/Appligator (Append Medical, Or Yehuda, Israel)	Implant-free LAA inversion and ligation	LAA inversion + suture ligation	N/A	U.S. early-feasibility IDE stage; APPLAUSE Study I (NCT07278869; IDE G240291) recruiting	
Cormos (Cormos Medical, Jena, Germany)	Plug or lobe–disc depending on configuration	J-shaped/multiple fixation hooks	Type I: ~18–28; Type II: 16–35	Premarket certification stage; regulatory approval in progress; not commercially available	

(**a**). Devices are grouped according to dominant design category and include platforms with current or previous regulatory certification. Regulatory status, geographical availability, and available size ranges may vary by region and should be interpreted according to the most recent manufacturer and regulatory information. FDA—Food and Drug Administration; CE—Conformité Européenne; NR—not reported. (**b**). Emerging devices are summarized according to design type, fixation mechanism, reported size range, and current stage of clinical development. Development status reflects the available clinical evidence and publicly reported trial or regulatory information at the search cut-off; for several devices, specifications and regulatory status remain incompletely reported or independently unverified. IDE—Investigational Device Exemption; LAAO—left atrial appendage occlusion; N/A—not applicable; NR—not reported; PFA—pulsed-field ablation.

**Table 5:** Anatomical features relevant to endocardial LAAO device selection and procedural planning [21,22,32,46]. Anatomical parameters should be interpreted together with preprocedural and intraprocedural imaging findings. Suggested device implications represent general design-based considerations rather than rigid selection rules.

Anatomical Feature	Relevance for LAAO Planning	Potential Device-Selection Implication
Ostial diameter, shape, and eccentricity	Determines the required sealing surface, disc, plug or umbrella sizing, and risk of incomplete ostial coverage	Large, oval, or highly eccentric ostia may favor devices with broader ostial coverage or dual-seal architecture
Landing-zone diameter	Determines device type and size	Sizing mismatch may increase the risk of residual leak, device instability, or excessive tissue stress
Usable appendage depth	Determines whether the selected device can be safely accommodated without distal wall contact or excessive compression	Shallow appendages may be less suitable for deeper intraluminal plug systems and may favor designs with ostial coverage or shorter implantation depth
Neck configuration and proximal angulation	Affects device type selection, delivery, coaxial alignment, deployment angle, and sealing orientation	Short, broad, or sharply angulated necks may complicate plug alignment and increase the risk of incomplete apposition or residual patency
Lobar anatomy and trabeculation	Influences anchoring, sealing completeness, residual appendage patency, and device–tissue interaction	Multilobulated or heavily trabeculated appendages may require broader sealing or distributed anchoring strategies, although comparative evidence remains limited
Thin wall/fragile appendage tissue	Relevant to perforation, pericardial effusion, tamponade, and anchoring-related trauma	Excessive radial force, oversizing, or aggressive anchoring should be avoided, particularly in shallow or sharply angulated appendages

**Table 6:** Major randomized and comparative studies of endocardial LAAO.

Study	Research Focus	Design	Population/Scope	Quantitative Results	Main Finding
PROTECT AF/PREVAIL pooled 5-year (2017)	LAAO vs. warfarin	Pooled analysis of two RCTs; *N* = 1114	NVAF; eligible for warfarin	- Primary composite: **HR 0.82** (**95% CI 0.58–1.17**) - Hemorrhagic stroke: **HR 0.20** (**95% CI 0.07–0.56**)	Comparable to warfarin for stroke/SE/CV or unexplained death; fewer hemorrhagic strokes and major bleeds.
Amulet IDE including longer follow-up (2021/2025)	Amulet vs. WATCHMAN 2.5	Device-comparison RCT; *N* = 1878	Device-comparison evidence	- Effectiveness: **2.8% vs. 2.8%; difference 0.00 pp** (**95% CI −1.55 to 1.55**)**; NI margin 3.2 pp** - LAA closure ≤ 5-mm residual jet: **98.9% vs. 96.8%**	Non-inferior to WATCHMAN 2.5 for safety/effectiveness; better LAA occlusion.
PRAGUE-17 long-term follow-up (2022)	LAAO vs. DOACs	RCT; *N* = 402	High-risk NVAF with prior cardioembolism and/or bleeding risk	- Primary composite: **sHR 0.81 (95% CI 0.56–1.18)** - ***p* = 0.006 for NI; NI margin HR 1.47**	Non-inferior to DOACs for composite CV, neurologic, and bleeding events.
SWISS-APERO (2022; longer follow-up 2024)	Amulet vs. WATCHMAN/FLX residual patency and leaks	Mechanistic imaging RCT; *N* = 221	Residual patency/leak imaging evidence	- Residual patency/crossover at 45 d: **67.6% vs. 70.0%** - **RR 0.97 (95% CI 0.80–1.16); *p* = 0.713**	Amulet was not superior to WATCHMAN/FLX for CT LAA patency; however leak patterns differed.
OPTION (2024/2025)	LAAO vs. oral anticoagulation after AF ablation	Post-ablation RCT; *N* = 1600	Post-ablation patients with elevated CHA_2_DS_2_-VASc score	- Death/stroke/SE: **5.3% vs. 5.8%; HR 0.91** (**95% CI 0.59–1.39**)**; *p* < 0.001 for NI** - Nonprocedural major/CRNM bleeding: **8.5% vs. 18.1%; HR 0.44** (**95% CI 0.33–0.59**)	Less nonprocedural major/CRNM bleeding; non-inferior to OAC for death/stroke/SE.
CHAMPION-AF (2026)	WATCHMAN FLX vs. NOACs as first-line strategy	First-line strategy RCT; *N* = 3000	OAC-eligible NVAF patients with elevated stroke risk	- CV death/stroke/SE: **5.7% vs. 4.8%; difference 0.9 pp** (**95% CI −0.8 to 2.6**)**; NI margin 4.8 pp** - Nonprocedural bleeding: **HR 0.55** (**95% CI 0.45–0.67**)	Non-inferior to NOACs for CV death/stroke/SE; less nonprocedural bleeding.
CLOSURE-AF (2026)	LAAO vs. physician-directed best medical therapy	High-risk RCT; *N* = 912	Older AF patients with high stroke and bleeding risk	- Primary composite: **HR 1.28** (**95% CI 1.01–1.62**) - ***p* = 0.44 for NI; NI margin HR 1.30**	Did not meet non-inferiority versus medical therapy for stroke/SE/major bleeding/CV or unexplained death.

The table summarizes pivotal trials and selected comparative evidence relevant to device performance, comparator therapy, and patient selection. AF—atrial fibrillation; CRNM—clinically relevant nonmajor; CT—computed tomography; CV—cardiovascular; DOAC—direct oral anticoagulant; LAA—left atrial appendage; LAAO—left atrial appendage occlusion; NOAC—non-vitamin K antagonist oral anticoagulant; NVAF—non-valvular atrial fibrillation; OAC—oral anticoagulation; RCT—randomized controlled trial; SE—systemic embolism.

**Table 7:** Ongoing and recently initiated studies in endocardial LAAO.

Study	Trial Goal/Comparator	Design/Sample Size	Population/Scope
VERITAS/Amulet 2 (Amulet 360) NCT06707688	Amulet 2 LAAO: safety/effectiveness	Single-arm device study *N* ≈ 400; active/not recruiting; early 45-day data available	NVAF patients undergoing LAAO with the next-generation Amulet platform
LAmbre Plus/REDUCE-AF NCT06465706	LAmbre Plus vs. commercial transcatheter LAAO devices	RCT Planned *N* ≈ 1826; active/not recruiting	NVAF patients eligible for percutaneous LAAO
GLACE/CLAAS AcuFORM NCT06580275	CLAAS AcuFORM under ICE guidance	Single-arm device study Planned *N* = 80; estimated completion 2026	NVAF patients undergoing LAA closure with CLAAS under ICE guidance
E-SeaLA first-in-human study NCT05731882	E-SeaLA: PFA + mechanical LAAO	First-in-human single-arm study Planned *N* = 10; recruiting/active in public listings	NVAF patients treated with simultaneous LAA mechanical closure and pulsed-field ablation
ENDOMATIC SEPIOLA NCT06099106	SEPIOLA clip-based LAA closure	Early feasibility single-arm study Planned *N* = 15; recruiting	NVAF patients at increased stroke risk seeking an alternative to long-term anticoagulation
SIMPLAAFY NCT06521463	WATCHMAN FLX Pro: aspirin or NOAC monotherapy vs. DAPT	Post-implant RCT Planned *N* = 1857; active/not recruiting in latest public listing	AF patients receiving WATCHMAN FLX Pro and eligible for study antithrombotic regimens
FADE-DRT NCT04502017	DAPT vs. genotype-guided therapy vs. low-dose DOAC	Post-implant RCT Planned *N* = 360; registry status inconsistent, last known recruiting/ongoing	Successful LAAO patients contraindicated or unsuitable for long-term OAC
SAFE-LAAC NCT03445949	30-day vs. 6-month DAPT; no therapy vs. SAPT after 6 months	Post-implant RCT with nonrandomized extension Planned *N* ≈ 200; estimated completion 2027	Patients after successful Amplatzer or WATCHMAN LAAO
LAAOS-4/LAAOS IV NCT05963698	WATCHMAN FLX/FLX Pro + OAC vs. OAC alone	RCT, PROBE; planned *N* = 4000; recruiting/ongoing	High-risk AF patients continuing long-term OAC

The table summarizes current research directions in LAAO, including next-generation devices, alternative closure technologies, combined ablation–closure strategies, procedural guidance, post-implant antithrombotic regimens, and adjunctive use of LAAO with oral anticoagulation. AF—atrial fibrillation; DAPT—dual antiplatelet therapy; DOAC—direct oral anticoagulant; ICE—intracardiac echocardiography; LAA—left atrial appendage; LAAO—left atrial appendage occlusion; NCT—National Clinical Trial number; NOAC—non-vitamin K antagonist oral anticoagulant; NVAF—non-valvular atrial fibrillation; OAC—oral anticoagulation; PFA—pulsed-field ablation; PROBE—prospective randomized open blinded endpoint; RCT—randomized controlled trial; SAPT—single antiplatelet therapy.

**Table 8:** Evidence-derived postprocedural antithrombotic strategies after LAAO.

Device/Strategy	Initial Therapy	Subsequent Therapy	Long-Term Therapy	Evidence/Status	Source(s)
WATCHMAN 2.5	VKA + aspirin (0–45 d)	DAPT (45 d–6 mo)	Aspirin	Pivotal RCT protocol	PROTECT AF [15]; PREVAIL [16]
WATCHMAN FLX	DOAC + aspirin (0–45 d)	DAPT (45 d–6 mo)	Aspirin	Prospective trial protocol	PINNACLE FLX [27]
Amulet	DAPT **or** aspirin + OAC (0–45 d)	DAPT (45 d–6 mo)	Aspirin	Pivotal RCT protocol	Amulet IDE [31]
Amulet	DAPT (0–6 mo) —		Antiplatelet therapy at physician discretion	FDA-labelled regimen	Amulet IFU [30]
LAmbre	DAPT (3–6 mo)	SAPT	SAPT, usually aspirin	Observational	[37,38,44]
PRAGUE-17	DAPT (0–3 mo)	SAPT	Aspirin	RCT protocol	PRAGUE-17 [14]
Low-dose DOAC	Apixaban 2.5 mg (twice daily) (0–3 mo)	Aspirin	Aspirin	Randomized emerging strategy	ADALA [54]
Short-term DOAC	DOAC (60 ± 15 d)	Protocol-dependent	Variable	Randomized emerging strategy	ANDES [55]
SAPT/no therapy	SAPT or no therapy	Variable/discontinuation	SAPT or no therapy	Observational	EWOLUTION [53]

VKA—vitamin K antagonist; DOAC—direct oral anticoagulant; DAPT—dual antiplatelet therapy, usually aspirin plus clopidogrel; SAPT—single antiplatelet therapy, usually aspirin; LAAO—left atrial appendage occlusion.

## Data Availability

No new data were created or analyzed in this study. Data sharing is not applicable to this article.
